# Functional and Reconstructive Outcomes of Square Flap in Postburn Axillary Contracture

**DOI:** 10.7759/cureus.98629

**Published:** 2025-12-07

**Authors:** Kawsar Ahmad, Shariff A Rahman, Tasnim Enam, Md. Shayedat-Ullah

**Affiliations:** 1 Plastic Surgery, National Institute of Burn and Plastic Surgery, Dhaka, BGD; 2 Pathology, Rajshahi Medical College Hospital, Rajshahi, BGD; 3 Endocrinology, National Institute of Burn and Plastic Surgery, Dhaka, BGD

**Keywords:** burn contracture release, complication, contracture band length, functional recovery, post-burn contracture, reconstructive outcomes, shoulder abduction, square flap

## Abstract

Background and aim

Post-burn axillary contracture (PBAC) represents a frequent and disabling sequela of burn injury, often impairing shoulder movement and hindering daily activities. Due to its promising functional and cosmetic outcome, the square flap technique has recently gained attention, yet data in the Bangladeshi context remain scarce. This study was planned to assess the functional and reconstructive outcomes of the square flap technique in PBAC reconstruction.

Methods

A prospective observational study was conducted at the National Institute of Burn and Plastic Surgery, Dhaka, from January 2023 to June 2024. Among the patients with type 1A and 1B PBAC, a total of 40 patients who were planned to undergo contracture release using the square flap technique were purposively included in the study. Patients presenting with unstable scars over the contracture site, presence of ulcers on the scar, skin malignancy at the contracture site, bony deformities of the affected joint, or recurrent contractures were excluded from the study. Preoperative and three-month postoperative assessments, such as shoulder abduction range, contracture band length, complications, and reconstructive outcomes, were recorded.

Results

The majority of the patients with post-burn contracture were adolescents with female predominance (65%). Most (80%) of the participants were suffering from type 1A axillary contracture, and flame burns were identified as the predominant cause, accounting for 92.5% of cases. The mean shoulder abduction improved significantly from 112° ± 20.15° preoperatively to 165° ± 17.03° postoperatively (p value: <0.001). Contracture band length increased from 4.68 ± 1.16 cm to 8.66 ± 1.40 cm (p value: <0.001). Tip necrosis (7.5%) and epidermolysis (10%) were the most common complications. The reconstruction outcome was good (82.5%).

Conclusion

Square flap reconstruction is an effective technique for releasing linear axillary contractures, providing excellent functional and cosmetic outcomes with minimal complications and a high level of patient satisfaction.

## Introduction

Burns continue to be among the most serious injuries encountered in emergency medicine, affecting both genders and all age groups worldwide, with Bangladesh being no exception [1,2]. For survivors, permanent scarring remains the most common and challenging long-term complication [3]. Postburn contracture significantly affects the quality of life for burn victims, as it is functionally debilitating, disfiguring, painful, and itchy, contributing to their overall morbidity [4]. The issue of post-burn contracture in the axilla presents significant challenges, particularly as shoulder mobility directly affects one's capacity to utilise the hand effectively for daily activities [5]. In moments of injury, it is common for the victim to exhibit a protective stance characterised by tightly adducted and flexed arms. Consequently, the injury typically adversely affects the anterior and posterior axillary folds, leaving the axillary fossa untouched [6]. Prevention stands as the most effective approach, encompassing early and consistent physical therapy (PT), stretching, and splinting [7] once a contracture has developed and become fixed, causing significant functional impairment, or has failed to respond to non-surgical treatments. Surgical options are considered the most powerful treatment for severe, established cases [8].

The defect should be repaired with donor tissues that have a similar texture, colour, and pliability. Skin flaps serve to replace scar tissues and mend the resulting defect following release, ultimately resulting in increased functional outcomes [4,6,9,10]. To facilitate operation and align with the characteristics of the skin, the preferred method for burn scar reconstruction is the use of adjacent skin flaps [11]. Various surgical techniques have been documented for the release of burn contractures. The Z-plasty technique stands as a time-honoured treatment method, effectively addressing the challenge of straight lines by enhancing length and reinstating the normal range of motion in joints [12,13]. Various techniques of Z-plasty exist, such as the four-flap, five-flap, and seven-flap methods. When applied at specific angles and lengths, these techniques can effectively assist in the release of scar contractures [12,13]. The square flap method, pioneered by Hyakusoku et al., serves as a highly effective technique for addressing post-burn contractures across various anatomical regions, including the axilla, cubital fossa, neck, digital contractures, perineum, and popliteal fossa [14-16]. Although it boasts exceptional surgical qualities for burn contracture release, its adoption among plastic surgeons remains limited. A study by Karki et al. revealed that the square flap technique is a dependable method for addressing mild to moderate axillary contractures in the anterior or posterior axillary folds, even in the presence of considerable adjacent scarring on the chest wall or back of types I and II [3]. Hifny et al. conveyed that the square flap method has been demonstrated as a straightforward technique that is easily replicable. The lengthening potential is commendable, and it proves effective in alleviating contracture bands upon long-term follow-up [12]. However, no such study was conducted from a Bangladeshi perspective, where burn injuries are a common public health problem with prevalent open flames and unsafe cooking practices. Considering its impact on upper limb mobility, daily functioning, and the ability to maintain independence, axillary contracture following burn injury presents a significant challenge - especially in settings with limited access to advanced microsurgical facilities. The square flap technique offers a practical, cost-effective, and reliable reconstructive solution. Its simplicity, reproducibility, and favourable functional outcomes make it particularly well-suited for broad application within the Bangladeshi healthcare system. Therefore, this study was undertaken to evaluate the functional (postoperative shoulder abduction) and reconstructive outcomes (including contracture band length and flap-related complications) following square flap reconstruction in post-burn axillary contracture patients.

## Materials and methods

A prospective observational study was conducted at the National Institute of Burn and Plastic Surgery (NIBPS), Dhaka, from January 1, 2023, to June 30, 2024. Among the patients with post-burn linear contractures involving the anterior axillary fold (type 1A) or posterior axillary fold (type 1B) [17], a total of 40 patients who were planned to be treated with the square flap technique were purposively included in the study. Exclusion criteria comprised patients with unstable scars over the contracture site, presence of ulcers on the scar, skin malignancy at the contracture site, bony deformities of the affected joint, or recurrent contractures. The aims and objectives of the study were discussed with the patients, and informed written consent was obtained. Ethical approval for the study was obtained from the Institutional Review Board of NIBPS (Memo no: SHNIBPS/ECC/2022/21). Prior to surgery, data were collected on sociodemographic characteristics (age, gender), burn etiology (flame burn, scald, electric burn), time since burn injury, type of prior burn management (at home, conservative, surgical), contracture type (type 1A or type 1B), range of shoulder abduction (in degrees using a goniometer), and length gain before contracture release. All procedures were performed by a team of trained plastic surgeons. A semi-structured questionnaire was developed and used for data collection.

Surgical technique (square flap) 

**Figure 1:**
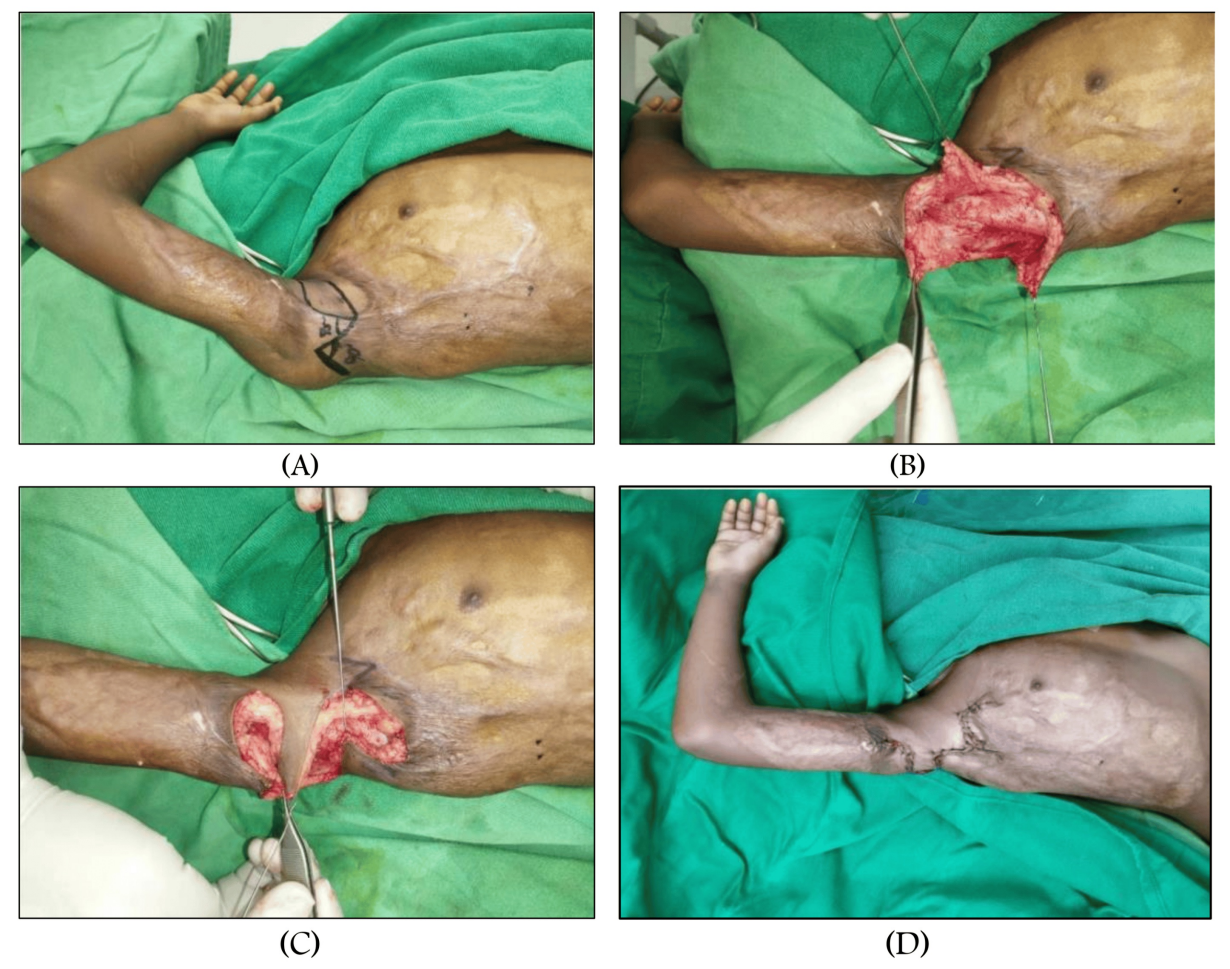
(A) Preoperative marking for the square flap technique. (B) Preoperative picture after raising the square flap and two transposition flaps. (C) Preoperative picture after flap advancement and transposition. (D) Postoperative suturing

The square flap procedure was performed in sequential steps. Initially, a standard square flap was designed, comprising a square advancement flap and two triangular transposition flaps with angles of 45° and 90°, respectively [3,11]. Local anaesthesia mixed with adrenaline (1:200,000 dilution in normal saline) was injected subcutaneously. Skin incisions were made along the marked lines using a No. 15 blade, followed by incision and dissection of the subcutaneous tissue. Electrocautery was used as needed. After releasing the contracture, the square flap was advanced, and the triangular flaps were transposed. Closure was achieved using 3-0 Vicryl for dermal sutures and 4-0 Prolene for superficial sutures (Figure 1).

Strategies to maintain flap vascularity

To reduce the risk of flap ischemia, various precautionary measures were implemented during the procedure. Careful manipulation of tissues was maintained during dissection to protect the subdermal plexus. The flap thickness was consistently maintained to prevent damage to the subcutaneous vascular network, crucial for flap perfusion. Electrocautery was used sparingly near the flap pedicles to mitigate the risk of thermal damage to perforators. Following flap elevation, the edges of the flap were examined for bleeding to ensure sufficient perfusion before inset. Undue tension on the flap during advancement and transposition was mitigated through meticulous planning of flap dimensions and, when necessary, additional subcutaneous release. Hemostasis was carefully achieved to prevent postoperative hematoma, which could jeopardise flap vascularity. Furthermore, the patient's limb was positioned to prevent flap stretching, and dressings were applied lightly to minimise the risk of pressure-related ischemia [18].

Postoperative management

Postoperative care followed a standardised protocol. The affected arm was maintained at 150° using a splint for three weeks (continuous for two weeks, followed by a night splint for one week) to protect the flap and prevent early recurrence of contracture, consistent with standard postoperative recommendations [19]. Adequate oxygenation, hydration, and nutrition were ensured to support wound healing. Oral antibiotics were administered according to hospital guidelines. Follow-up assessments were conducted on the 5th, 14th, and 30th postoperative days to monitor for wound infection, epidermolysis, tip necrosis, and general wound healing. Sutures were removed at the end of the second postoperative week. Complications were managed based on severity: epidermolysis was treated with secondary healing, and tip necrosis was debrided and allowed to heal secondarily. Patients with uneventful recovery were discharged around the fifth postoperative day, while others stayed longer depending on complications. A final assessment was conducted at the three-month follow-up, including photographic documentation.

Outcome assessment

At three months postoperatively, outcome assessment included the evaluation of shoulder abduction range (in degrees using a goniometer), the length gained following contracture release, and the presence of complications such as wound infection, flap epidermolysis, tip necrosis, wound dehiscence, and hypertrophic scarring. The reconstructive outcome of the flap was classified as good, satisfactory, or poor, according to the criteria described by Islam et al. in 2019. Flap outcomes were evaluated based on two predefined criteria: flap survival and the presence or management of flap-related complications. Outcomes were classified as good when there was complete flap survival with no associated complications. A satisfactory outcome was defined by the presence of marginal or partial flap loss and/or flap-related complications that could be managed conservatively without surgical intervention. Outcomes were considered poor in cases of total flap loss or when flap-related complications required surgical management. Classification was assigned only when both conditions of a category were fulfilled.

Data analysis

The statistical analyses were carried out using the Statistical Product and Service Solutions (SPSS, version 23; IBM SPSS Statistics for Windows, Armonk, NY). Continuous data were summarised using mean and standard deviation, minimum, and maximum. The data's normal distribution was assessed using the Shapiro-Wilk test. A paired t-test was done to determine the changes in abduction range and length gain from baseline to three months following surgical treatment. To determine the association between categorical variables, chi-square tests were done. A p-value of <0.05 was considered to indicate statistical significance.

## Results

The mean age of the participants was 17.02 ± 10.02 years. The majority (60.0%) were aged ≤15 years, followed by 25.0% in the 16-30 years group and 15.0% in the 31-45 years group, indicating that participants were predominantly adolescents. In terms of gender distribution, females were more represented (65.0%) compared to males (35.0%), indicating a female predominance in the study population (Table 1).

**Table 1:** Sociodemographic profile of the patients with post-burn axillary contracture (n=40) SD: standard deviation Data were presented as frequency (percentage), mean±SD

Sociodemographic characteristics		Frequency (percentage)
Age (years)	≤15	24 (60.0)
	16-30	10 (25.0)
	31-45	6 (15.0)
	Mean±SD	17.02±10.02
Gender	Male	14 (35.0)
	Female	26 (65.0)

Among the 40 patients with post-burn axillary contractures, the majority sustained flame burns (92.5%), followed by scalds (5.0%) and electrical burns (2.5%). More than half of the patients (55.0%) presented more than 12 months after the initial burn injury, while 45.0% sought treatment within 12 months. The time elapsed since the burn injury ranged from five to 60 months, with a mean duration of 18.9 ± 12.65 months. Regarding initial burn management, 27.5% were treated at home, 50.0% received conservative care, and 22.5% underwent surgical intervention. The most common type of axillary contracture was type 1A (80.0%), with type 1B observed in 20.0% of cases. The left side was more commonly affected (67.5%) compared to the right side (32.5%) (Table 2).

**Table 2:** Distribution of burn etiology, management, and contracture characteristics among study participants SD: standard deviation Data were presented as frequency (percentage), mean±SD, minimum, maximum

Burn etiology, management, and contracture		Frequency (percentage)
			Etiology of burn	Flame Burn	37 (92.5)
Scald	2 (5.0)		
Electric burn	1 (2.5)		
Time passed since burn injury	≤12 (months)	18 (45.0)
	>12 (months)	22 (55.0)
	Mean ±SD	18.9 ±12.65
	Minimum-maximum	5- 60
Management of burn	At home	11 (27.5)
	Conservative	20 (50.0)
	Surgical	9 (22.5)
Type of axillary contracture	Type 1A	32 (80.0)
	Type 1B	8 (20.0)
Side of axillary contracture	Right	13 (32.5)
	Left	27 (67.5)

A significant improvement was observed in both the range of abduction and the contracture band length following surgical intervention using the square flap technique. The mean preoperative range of abduction was 112 ± 20.15 degrees, which increased to 165 ± 17.03 degrees postoperatively. Similarly, the mean contracture band length improved from 4.68 ± 1.16 cm before surgery to 8.66 ± 1.40 cm after the procedure. These changes were found to be statistically significant (p value: <0.001), indicating the effectiveness of the square flap technique in releasing axillary contractures and enhancing shoulder mobility (Table 3).

**Table 3:** Comparison of pre- and postoperative outcomes following square flap reconstruction (n=40) A paired t-test was done. Data were expressed as mean±SD.

Variables	Preoperative	Postoperative	p-value
Range of abduction (degree)	112±20.15	165±17.03	<0.001
Contracture band length (cm)	4.68±1.16	8.66±1.40	<0.001

The contracture band length increased by 31.54% to 185.0% across patients, with a median improvement of 87.5%, indicating a substantial postoperative length gain. The shoulder joint abduction increased by 20% to 87.50% across patients, with a mean improvement of 50.24%, reflecting a considerable postoperative enhancement in shoulder mobility (Table 4).

**Table 4:** Postoperative functional and structural improvement following square flap release Data were expressed as mean, median (range).

Variables	Mean	Median (range)
Percentage of improvement in shoulder joint abduction	50.24%	50.0% (20.0%-87.50%)
Percentage of improvement in contracture band length	93.26%	87.5% (31.54%-185.0%)

**Figure 2:**
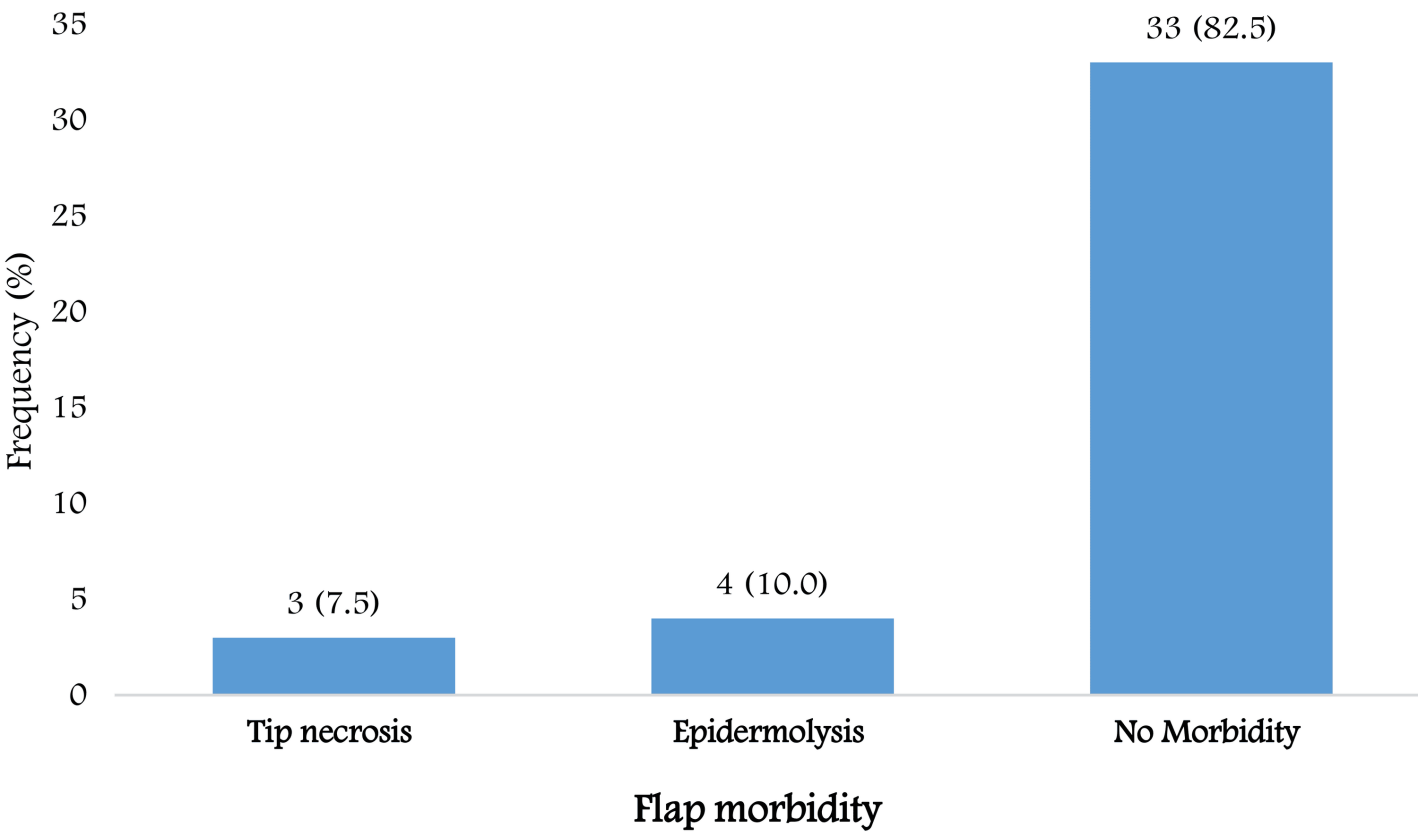
Distribution of flap morbidity following square flap reconstruction

Flap morbidity was observed in seven patients (17.5%). Of these, three patients (7.5%) experienced tip necrosis, and four patients (10%) developed epidermolysis. No flap morbidity was observed in 33 patients (82.5%) (Figure 2).

**Figure 3:**
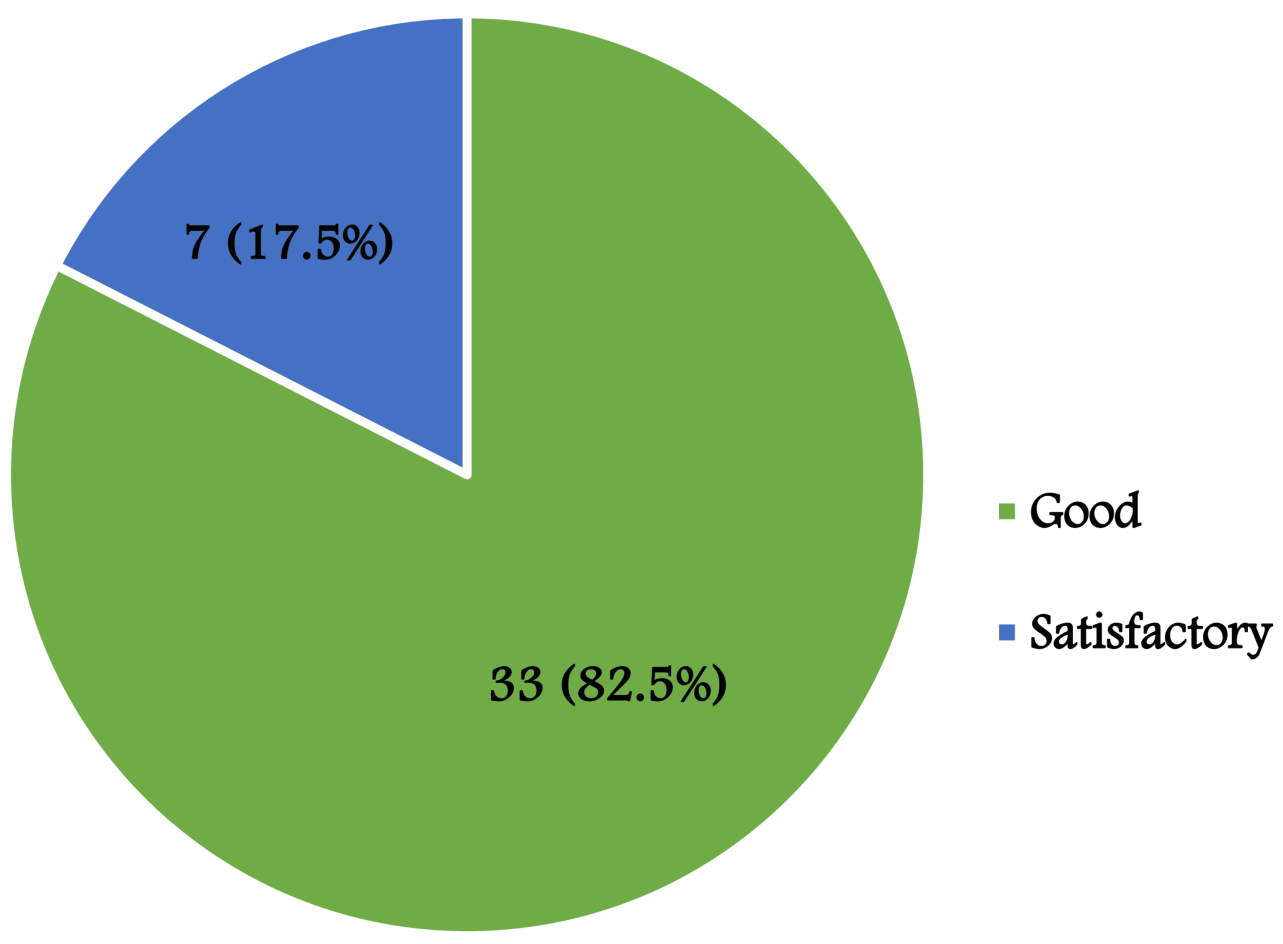
Postoperative reconstruction outcomes using the square flap technique

Reconstruction outcomes following the square flap technique were classified as good in 33 patients (82.5%) and satisfactory in seven patients (17.5%) (Figure 3).

## Discussion

Post-burn axillary contracture is frequently observed after severe burn insult and is usually accompanied by a scarred adjacent area [20]. The primary goal of surgical procedures for treating post-burn axillary contracture is to release the scar tissue and restore optimal joint function. Locally displaced flaps commonly used for this purpose include Z-plasty, YV plasty, W-plasty, four- or five-flap plasty, and the square flap technique. Among these, Z-plasty is a conventional method that effectively corrects linear contractures, lengthens the affected tissue, and helps reinstate the normal range of joint motion [14,21]. However, recently, the square flap technique has gained considerable attention due to its benefits in enhancing mobility, improving daily functioning, and enabling patients to maintain independence [16]. In the context of our country, this study was undertaken to evaluate the effectiveness of the square flap procedure, and it was found that it significantly improves shoulder joint abduction and contracture release length, with minimal or negligible complications, and yields high patient satisfaction.

As burn outcomes and survival rates have improved, the goal of managing major burns has changed from just making sure the burn survivor survives to making sure they are as functional and aesthetically pleasing as possible. Because of its unique anatomical features and functional demands, the axilla is particularly susceptible to contracture formation and remains a difficult site to repair [22]. The square flap technique was used to treat all patients in this study. Preoperatively, the mean range of shoulder abduction was 112 ± 20.15°, which improved to 165 ± 17.03° postoperatively. The shoulder joint abduction improved by 20% to 87.50% among patients, with a mean enhancement of 50.24%, indicating a significant postoperative increase in shoulder mobility. In their series of 13 axillary contractures treated with a square flap, Huang et al. reported a preoperative abduction of 86.15° and a postoperative abduction of 152.3°, achieving a 76.78% improvement [23]. Karki et al. [3] reported an even greater improvement of 218.5% with the square flap, which was higher than the results obtained with multiple Z-plasties, parascapular flaps, or split-thickness skin grafts [19]. Hifny et al. also found significant postoperative improvement in abduction using the square flap, with results comparable to those of the five-flap Z-plasty [24]. Overall, the improvement achieved with the square flap is remarkable and, in some cases, superior to other techniques. This improvement is most likely due to the method’s ability to restore full skin length in multiple directions while preserving vascularity and reducing tension, which together enable near-normal joint movement postoperatively [3,23]. The square flap, therefore, represents a strong alternative to the five-flap Z-plasty in the management of axillary contractures [24].

In this study, the contracture band length increased by 31.54-185.0%, with a median gain of 87.5%, indicating meaningful postoperative improvement. Although the percentage increase was lower than that reported by Hifny, who observed 212-350% elongation (average 247%) [12], the postoperative lengthening achieved in our cohort still demonstrates effective contracture release and functional restoration using the square flap technique. This finding aligns with Hifny et al., who demonstrated that length gain with the square flap was significantly greater than that achieved with the five-flap Z-plasty [24]. Huang et al. further reinforced this advantage, reporting that the square flap could lengthen the original scar band by 2.825-fold, surpassing the ≤2.239-fold elongation attainable with single, four-flap, or five-flap Z-plasties [23]. Beyond quantitative gains, the square flap offers distinct anatomical and physiological benefits, including lower physiological tension across the repair site and preservation of a robust vascular supply [23].

Complication rates reported in the literature vary. Karki et al. documented an 18.8% incidence, including tip necrosis, graft loss, and re-contracture [19], whereas Sarker et al. reported complications in only 9.5% of cases [25]. In the present series, flap morbidity occurred in seven patients (17.5%): tip necrosis in three patients (7.5%) and epidermolysis in four patients (10%). Importantly, these events did not compromise the overall reconstructive goals, with outcomes rated as satisfactory in terms of both function and appearance.

The appeal of the square flap lies in its simplicity, reproducibility, and versatility. By advancing a well-vascularized, pliable block of tissue into the defect, the technique not only delivers substantial lengthening but also disrupts the linear scar line - reducing the risk of recurrence. When coupled with its favourable cosmetic profile on long-term follow-up [12], the square flap stands out as an elegant, pragmatic solution for axillary contracture reconstruction. However, postoperative rehabilitation is essential to maximise functional recovery following square flap reconstruction for axillary contracture.

Limitations

Outcomes were assessed at three months postoperatively; however, a longer follow-up period would provide a clearer understanding of long-term results. The absence of a comparative group limits the ability to directly contrast the square flap with other reconstructive techniques. Moreover, the use of a subjective scale to evaluate reconstructive outcomes introduces the potential for bias.

## Conclusions

This study confirms that the square flap technique effectively improves shoulder joint abduction and contracture band length in mild-to-moderate post-burn axillary contracture patients, achieving significant functional gains with minimal complications. The procedure was well-tolerated, with satisfactory cosmetic outcomes and high patient satisfaction. Its simplicity and reproducibility make it a valuable reconstructive option. Nonetheless, the study’s relatively short follow-up period and lack of a comparative group suggest the need for longer-term, controlled studies to further validate these findings and establish the square flap as a preferred technique in axillary contracture management.
